# The C30-Modulation of Betulinic Acid Using 1,2,4-Triazole: A Promising Strategy for Increasing Its Antimelanoma Cytotoxic Potential

**DOI:** 10.3390/molecules27227807

**Published:** 2022-11-12

**Authors:** Gabriela Nistor, Marius Mioc, Alexandra Mioc, Mihaela Balan-Porcarasu, Roxana Racoviceanu, Alexandra Prodea, Andreea Milan, Roxana Ghiulai, Alexandra Semenescu, Cristina Dehelean, Codruța Șoica

**Affiliations:** 1 Department of Pharmaceutical Chemistry, Faculty of Pharmacy, “Victor Babes” University of Medicine and Pharmacy Timisoara, Eftimie Murgu Square No. 2, 300041 Timişoara, Romania; 2 Research Centre for Pharmaco-Toxicological Evaluation, “Victor Babes” University of Medicine and Pharmacy, Eftimie Murgu Sq., No. 2, 300041 Timisoara, Romania; 3 Department of Anatomy, Physiology, Pathophysiology, Faculty of Pharmacy, Victor Babes University of Medicine and Pharmacy, 2nd Eftimie Murgu Sq., 300041 Timisoara, Romania; 4 Institute of Macromolecular Chemistry ‘Petru Poni’, 700487 Iasi, Romania; 5 Department of Pharmacology-Pharmacotherapy, Victor Babes University of Medicine and Pharmacy, 2nd Eftimie Murgu Sq., 300041 Timisoara, Romania; 6 Department of Toxicology, Faculty of Pharmacy, “Victor Babes” University of Medicine and Pharmacy, Eftimie Murgu Sq., No. 2, 300041 Timisoara, Romania

**Keywords:** betulinic acid, triterpenes, triazole, apoptosis, cell viability, high-resolution respirometry

## Abstract

Cancer, in all its types and manifestations, remains one of the most frequent causes of death worldwide; an important number of anticancer drugs have been developed from plants, fungi and animals, starting with natural compounds that were later derivatized in order to achieve an optimized pharmacokinetic/pharmacological profile. Betulinic acid is a pentacyclic triterpenic compound that was identified as an anticancer agent whose main advantage consists in its selective activity, which ensures the almost total lack of cytotoxic side effects. Conjugates of betulinic acid with substituted triazoles, scaffolds with significant pharmacological properties, were synthesized and tested as anticancer agents in order to achieve new therapeutic alternatives. The current paper aims to obtain a C30-1,2,4-triazole derivative of betulinic acid simultaneously acetylated at C3 whose biological activity was tested against RPMI melanoma cells. The compound revealed significant cytotoxic effects at the tested concentrations (2, 10 and 50 μΜ) by significantly decreasing the cell viability to 88.3%, 54.7% and 24.5%, respectively, as compared to the control. The compound’s testing in normal HaCaT cells showed a lack of toxicity, which indicates its selective dose-dependent anticancer activity. The investigation of its underlying molecular mechanism revealed an apoptotic effect induced at the mitochondrial level, which was validated through high-resolution respirometry studies.

## 1. Introduction

Cancer, in all its types and manifestations, remains one of the most frequent causes of death worldwide, disputing the top position with heart diseases; despite tremendous progress that significantly diminished mortality rates, the WHO’s International Agency for Research on Cancer estimates that approximately 9.6 million new cancer cases will emerge until 2040. Drug development in cancer has hugely evolved from accidental discovery (i.e., nitrogen mustards) to drugs designed according to various strategies resulting from studying the empirical biological data. An important number of anticancer drugs have been developed from plants, fungi and animals, starting with the natural compounds that were later derivatized in order to achieve an optimized pharmacokinetic/pharmacological profile. Betulinic acid (BA) was identified by Pisha et al. as an antimelanoma agent in 1995 [1]; it is a pentacyclic triterpenic compound abundantly found in many plants—in particular, Betula spp.—and displays numerous biological activities, such as anticancer [2], anti-inflammatory [3], antiviral [4], antidiabetic [5] and antiparasitic, [6] as well as other beneficial effects on multiple organs. Its main advantage consists in its selective anticancer activity, which ensures the almost total lack of cytotoxic side effects. In terms of molecular mechanisms, it was established that the compound mainly acts by causing a direct mitochondrial cell apoptosis, thus bypassing resistance to conventional chemotherapy drugs [7]. Since data reported by Pisha et al., numerous papers published a huge amount of information regarding betulinic acid’s pharmacokinetic and pharmacodynamic properties. Its main disadvantage consists in its low water solubility, which presumably relates to its low bioavailability. Therefore, two directions were approached in order to improve its drug-ability: the synthesis of new derivatives and the preparation of optimized pharmaceutical formulations. The chemical modulations focused on several key positions in betulinic acid’s molecule: C3—hydroxyl, C28—carboxyl, ring A and the C20-C29 double bond. These derivatizations lead to a large collection of compounds with triterpenic scaffolds and higher biological effects compared to their parent compounds [8]. In addition, hybrids of betulinic acid with functional moieties were obtained that either have the ability to target specific organelles, such as mitochondria, or to ensure the release of higher amounts of nitric oxide. Both processes are directly correlated to their cytotoxicity. Moreover, the combination of betulinic acid with other biologically active molecules leads to bioconjugates that are able to preserve the properties of both parent compounds. Nitrogen-containing heterocyclic compounds are one of the main building blocks in medicinal chemistry, representing 60% of the small-molecule drugs collected in various databases [9]. Among these compounds, triazoles stand as scaffolds with superior pharmacological properties. Both 1,2,3- and 1,2,4-triazoles have the ability to react with electrophilic and nucleophilic agents due to their structural characteristics, thus providing the potential to develop new biologically active entities [10]. Conjugates of betulinic acid with substituted triazoles at C30 and various modulations of the C3 hydroxyl were synthesized, leaving the C28-carboxyle group intact. The triazole conjugate bearing a benzaldehyde moiety exhibited the smallest IC_50_ value (3.3 μM) and acted in a similar manner with actinomycin D [11]. Khan et al. synthesized a series of 1,2,3-triazole derivatives of BA, among which the 28{1*N* (4-fluoro phenyl)-1*H*-1, 2, 3-triazol-4-yl} methyloxy betulinic ester was emphasized as more potent than the parent compound with IC50 values of 5–7 μM. In addition, the authors revealed that the compound exerted its anticancer effect in human leukemia cells through intrinsic and extrinsic apoptosis induction [12]. Another triazole derivative, 30-[4-(4-fluorophenyl)-1*H*-1,2,3-triazol-1-yl] betulinic acid, showed a very low IC_50_ value (1.3 μM) against the same HL-60 leukemia cell-line [13]. Triazole moieties have also been used as linkers for the synthesis of betulinic acid-AZT bioconjugates at C2 [14] but also at C28 through the derivatization of the carboxyl group [15].

The current paper proposes the synthesis and biological assessment of a novel C30 BA-1,2,4-triazole derivative in order to gain biological-structural insights regarding the antimelanoma effect of these types of structures.

## 2. Results

### 2.1. Chemistry

**Figure 1:**
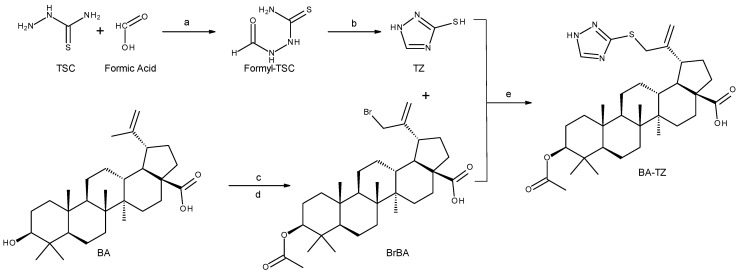
Synthesis pathways used to obtain 3β-*O*-Acetyl-30-(1H-1,2,4-triazole-3-ylsulfanyl)-betulinic acid (BA-TZ); TSC = thiosemicarbazide, TZ = 1,2,4-triazole-3-thiol, BA = betulinic acid, BrBA = 3β-*O*-Acetyl-30-bromo-betulinic acid; reaction conditions: a. reflux, 30 min; b. H_2_O, NaOH, reflux, 1h; c. acetic anhydride, pyridine, DMAP, r.t, 12h; d. NBS, CCl_4_, r.t, 48h; e. DMF, K_2_CO_3_, r.t, 72 h.

Synthesis schemes and reaction conditions used to obtain 3β-*O*-Acetyl-30-(1*H*-1,2,4-triazole-3-ylsulfanyl)-betulinic acid (BA-TZ), are depicted in Figure 1. Both 1,2,4-triazole-3-thiol (TZ) and the brominated acetyl-BA (BrBA) were obtained in good yields (>50%) using variations of previously reported similar reactions [16]. The alkylation of TZ’s thiol group using BrBA in DMF/K_2_CO_3_ produced lower yields of BA-TZ as compared to previous steps. While the precursors were relatively pure, the reaction produced at least two additional major components, as revealed by TLC analysis. Nevertheless, the high Rf values differences made the chromatographic separation (chloroform: ethylacetate 1:1 eluent) affordable, while future experiments for the determination of the chemical identity of the other quantitative relevant products were not performed at this time. All spectral data are available in the Supplementary Materials file submitted with this article.

### 2.2. The Effect of BA-TZ on the Viability of Normal and Malignant Melanoma Cell Lines

**Figure 2:**
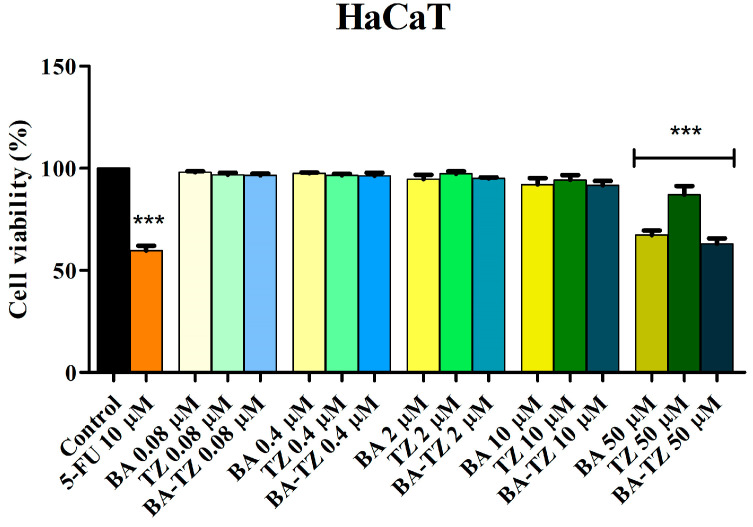
Cell viability of HaCaT cells after 24 h treatment with BA-TZ 0.08, 0.4, 2, 10 and 50 μM, determined using the MTT assay. Untreated cells were used as the negative control, whereas 5-FU was used as the positive control. The results are expressed as cell viability percentage (%) normalized to the negative control (100%). The data represent the mean values ± SD of three independent experiments performed in triplicate. The statistical differences vs. the negative control was determined using two-way ANOVA analysis followed by Bonferroni’s multiple comparisons post-test (*** *p* < 0.0001).

BA-TZ cytotoxic activity on HaCaT and RPMI-7951 cell lines was analysed using the MTT assay (3-(4,5-dimethylthiazol-2-yl)-2,5-diphenyltetrazolium bromide) after a 24 h treatment period with various concentrations (0.08, 0.4, 2, 10 and 50 μM). As shown in Figure 2, exposure of normal HaCaT cell lines to BA-TZ in concentrations up to 10 μM did not significantly impact cell viability, whereas the positive control 5-fluorouracil (5-FU) at 10 μM decreased cell viability at 59.66% vs. control. Similar results were obtained when BA and TZ alone were tested at the same concentrations on the HaCaT cell line (Figure 2). However, at the highest concentration (50 μΜ), all tested compounds (BA, TZ and BA-TZ) significantly decreased cell viability vs. control (67.3%, 87.5% and 63.2%).

**Figure 3:**
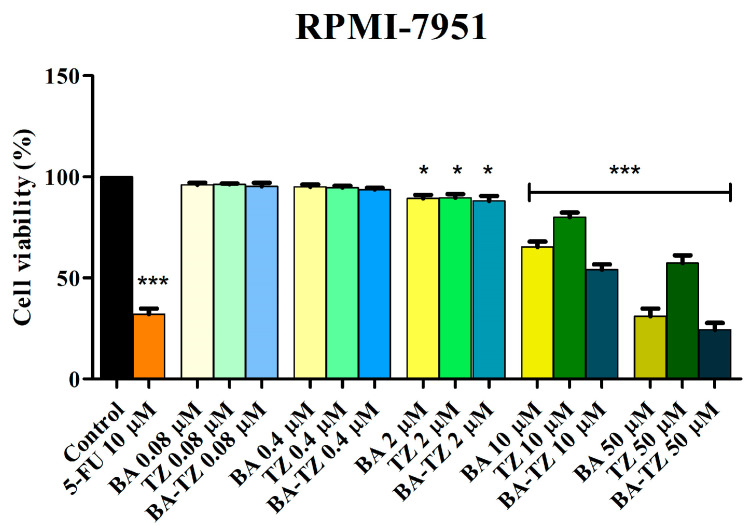
Cell viability of RPMI-7951 cells after 24 h treatment with BA-TZ 0.08, 0.4, 2, 10 and 50 μM, determined using the MTT assay. Untreated cells were used as negative control, while 5-FU was used as positive control. The results are expressed as cell viability percentage (%) normalized to the negative control (100%). The data represent the mean values ± SD of three independent experiments performed in triplicate. The statistical differences vs. the negative control was determined using two-way ANOVA analysis followed by Bonferroni’s multiple comparisons post-test (* *p* < 0.05 and *** *p* < 0.0001).

In the malignant melanoma RPMI-7951 cell line, BA-TZ at concentrations of 2, 10 and 50 μΜ significantly decreased cell viability to 88.3%, 54.7% and 24.5%, as compared to the negative control (100%). At the same time, 5-FU, at the tested concentration (10 μΜ), decreased cell viability down to 32%. (Figure 3). Significant decreases in cell viability were also obtained when BA and TZ were tested alone at the same concentrations, but both to a lesser degree compared with BA-TZ. Against the negative control, BA decreased cell viability to 89.1%, 65.1% and 31.3% when tested at 2, 10 and 50 μΜ, whereas TZ only to 89.9%, 80.2% and 57.4% (Figure 2).

### 2.3. Morphological Observation of RPMI-7951 Cells Using DAPI Staining

**Figure 4:**
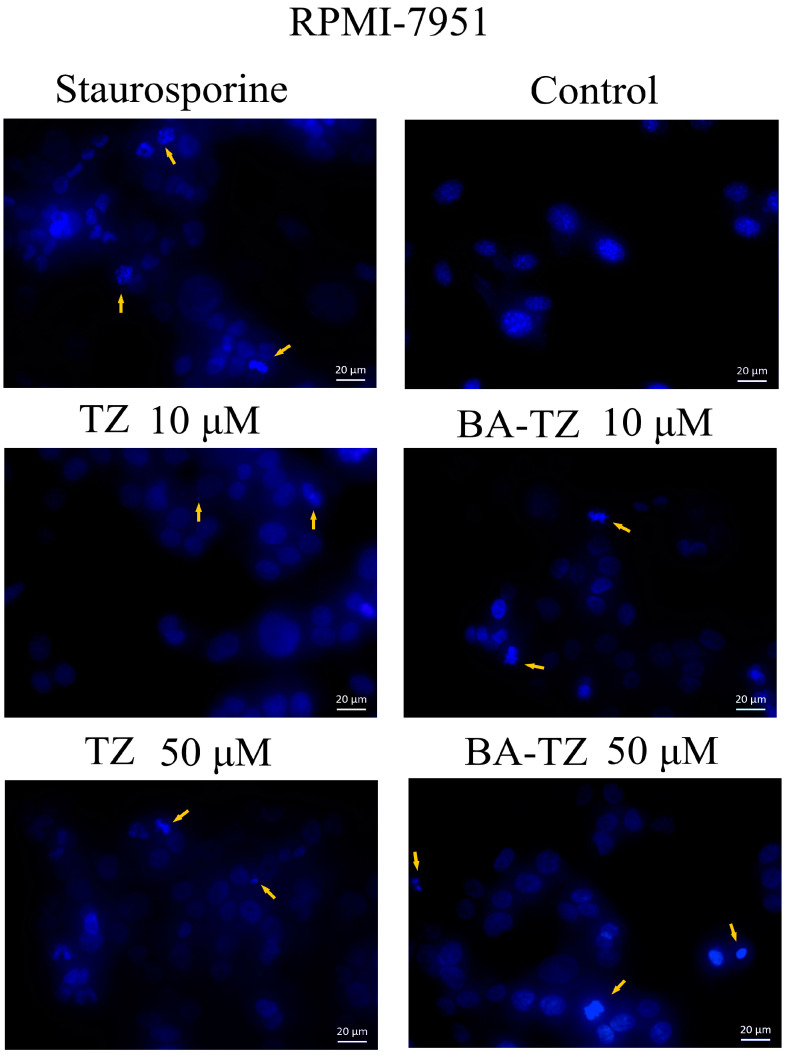
Morphological observation of RPMI-7951 cells using DAPI staining, after treatment with TZ and BA-TZ at 10 and 50 μM for 24 h. The staurosporine solution (5 μM) was used as positive control for apoptotic changes at the nuclear level. The yellow arrows represent signs of apoptosis, such as nuclear shrinkage, condensation, fragmentation and cellular membrane disruption.

Due to the high antiproliferative activity observed for BA-TZ at 10 and 50 μM on the RPMI-7951 cell line, these concentrations were selected in order to further investigate the compound’s more intimate cytotoxic mechanism by using the DAPI staining assay. Considering the insufficient literature data regarding TZ’s effect and mechanism against melanoma cell lines, 10 and 50 μM of TZ were also tested on RPMI-7951 cells using DAPI staining. Following this procedure, signs of apoptosis, such as nuclei condensation and shrinkage, were revealed for both compounds at 10 μM (yellow arrow, Figure 4). Besides nuclei condensation/shrinkage, nuclear fragmentation, another sign of apoptosis, was recorded upon testing TZ and BA-TZ in the highest concentration (50 μM) (Figure 4).

### 2.4. Evaluation of Mitochondrial Function Using High-Resolution Respirometry

**Figure 5:**
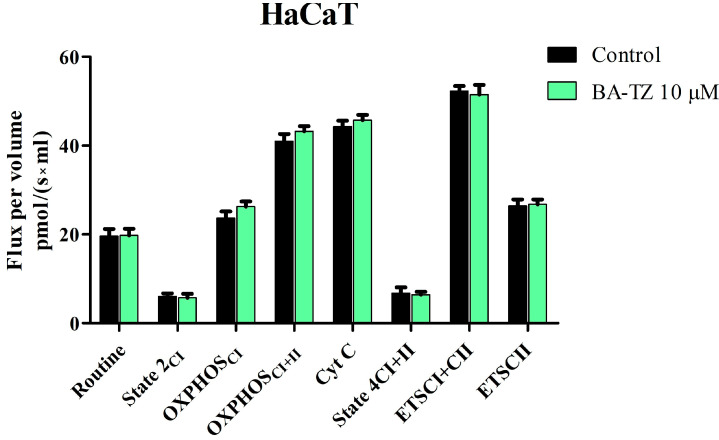
Mitochondrial respiratory rates of permeabilized HaCaT cells following 24 h treatment with BA-TZ 10 μM. Data represent the mean ± SD of five individual experiments. The statistical differences vs. control was determined using one-way ANOVA analysis followed by Tukey’s multiple comparisons post-test. Values with *p* < 0.05 were considered to have a statistically significant difference (*p* > 0.5 no significant differences).

**Figure 6:**
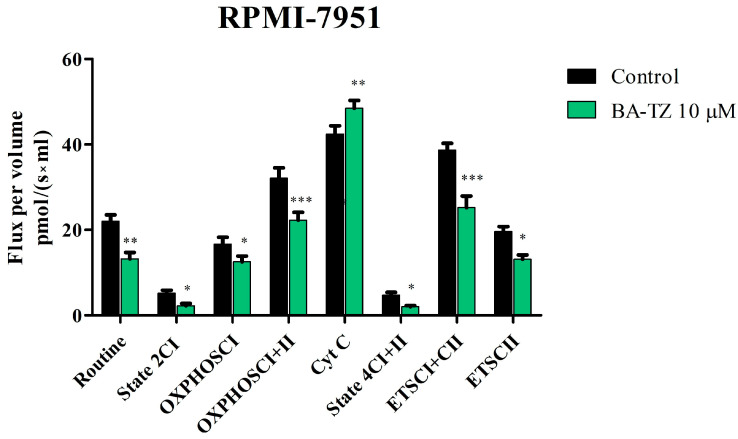
Mitochondrial respiratory rates of permeabilized RPMI-7951 cells following 24 h treatment with BA-TZ 10 μM. Data represent the mean ± SD of five individual experiments. The statistical differences vs. control was determined using one-way ANOVA analysis followed by Tukey’s multiple comparisons post-test. Values with *p* < 0.05 were considered to have a statistically significant difference (*p < 0.05, ** *p* < 0.01 and *** *p* < 0.001).

The high-resolution respirometry study of mitochondrial respiration showed that, at 10 μM, BA-TZ significantly inhibits mitochondrial function of RPMI-7951 cells, without affecting normal HaCaT cells (Figure 5 and Figure 6). BA-TZ treatment decreased the routine respiration (13.22 vs. control 22.03), the basal respiration (2.22 vs. control 5.17) and LEAK respiration (2.04 vs. control 4.73) of RPMI-7951 cells (Figure 6). In the same cell line, the active respiration dependent both on complex I alone (OXPHOS_CI_) and on complex I + complex II (OXPHOS_CI+II_) was also decreased as a result of BA-TZ application vs. control: (12.5 vs. 17.08 and 22.2 vs. 32.1). ETS_CI+II_ and ETSC_II_ also decreased significantly following BA-TZ treatment (25.20 and 13.1) vs. control (38.69 and 19.61). Interestingly, the only mitochondrial rate increased by BA-TZ treatment was Cyt c, with a Flux per volume pmol/(s×ml) of 49.6 vs. control 41.9 (Figure 6).

## 3. Discussions

Betulinic acid derivatives can be described as organic compounds with low molecular weight synthesized by various paths from the plant-derived natural product. Depending on the side-chain position and specific structure, various semisynthetic derivatives can exert different pharmacological activities. Most derivatives were tested as anticancer agents, but the antiviral effect was identified as well [17]. The introduction of a polar triazole ring on the triterpenic scaffold induced the ability to form hydrogen bonds, thus improving the overall water solubility of the semisynthetic derivative. Subsequently, an optimization in its bioavailability due to the hydrophilic character of the biological environment occurred. In effect, by altering the hydrogen bonding ability and polarity of the molecules, the triazole moiety is able to improve their physicochemical properties, toxicology, pharmacokinetics and pharmacology [18]. It is possible that the triazole moiety becomes part of the pharmacophore fragment of the resulting compound.

In the current study, we were able to synthesize a 1,2,4-triazole derivative of betulinic acid, the heterocycle being grafted in the C30 position of the triterpenic scaffold. The C28-carboxyle remained free, whereas the C3-hydroxyl was acetylated during synthesis and tested as such. To the best of our knowledge, only the 1,2,3-triazole ring was used as a substitute of the C30 position in the molecule of betulinic acid through the involvement of click chemistry [11]. No compound with the 1,2,4-triazole moiety in this position was previously reported. The compound was synthesized with the dual purpose of improving betulinic acid’s cytotoxic activity and optimizing its pharmacokinetic profile through the presence of a triazole moiety. The biological testing started with the assessment of cell viability by means of an MTT test conducted in RPMI-7951 melanoma cells. The MTT dye is a widely used, commercially available redox indicator that can evaluate cell metabolic activity and overall health status [19]. BA-TZ was applied on cancer cells for 24 h in five concentrations. Only the three higher concentrations, 2, 10 and 50 μM, induced a significant decrease in cell viability in a dose-dependent manner compared to control. The two individual compounds, BA and TZ, were also tested against the same cell line, exhibiting noticeable cytotoxic effects but inferior to their semisynthetic hybrid.

Shi et al. synthesized a large series of C30 1,2,3-triazole-substituted BA derivatives and tested their cytotoxic activities against HL-60 leukemia cells. Most of the newly obtained derivatives displayed higher bioactivity compared to the parent compound, BA [13]. The authors reported that larger side chains with lipophilic or aromatic side chains favored an increased cytotoxicity. Our results are in line with these findings, which also suggest that future research should focus on the introduction of supplementary groups on the triazole ring in order to further increase the compounds’ cytotoxicity. A second study [11] that introduced variously substituted triazole moieties in BA’s C30 position revealed that acetylation of the C3-hydroxyl group induced higher cytotoxic activity compared to compounds bearing the free group. The authors concluded that the conjugation of C30 with large triazole fragments acts as an important pharmacophore, being responsible for the cytotoxic effect. The same study attributed the specific bioavailability of each compound to the functional group of C3. In this regard, compounds with both C3 and C28 free hydroxyls display two hydrophilic groups on opposite sides of the molecule that might prejudice the compound’s cell membrane permeability. The introduction of the acetyl moiety with its small size and lipophilic character presumably counteracts the hydrophilic effect, therefore improving membrane permeability. Therefore, it is presumable that, in our case, the presence of the C3-acetylated hydroxyl contributes to the overall increased cytotoxicity of the triazole derivatives by increasing its membrane permeability.

In order to investigate its selectivity, BA-TZ cytotoxic activity in HaCaT cell lines was also analyzed using the MTT assay by a 24 h treatment period with various concentrations (0.08, 0.4, 2, 10 and 50 μM). Exposure of normal HaCaT cells to BA-TZ in low concentrations (≤10 μM) did not significantly impact cell viability, thus revealing a lack of toxicity. Similar results were obtained when BA and TZ were tested alone at the same concentrations in HaCaT cells. Only the highest concentrated samples (50 μΜ) from all tested compounds (BA, TZ and BA-TZ) significantly decreased cell viability vs. control (67.3%, 87.5% and 63.2%). Corroborating the results in both normal and malignant melanoma cells, one can state that the newly synthesized BA-TZ exhibits a selective cytotoxic activity when applied in 2 and 10 μM samples. The 50 μM sample, similarly to 5-FU tested at 10 μM, although cytotoxic against melanoma cells, lacks the level of selectivity shown by the two lower concentrations. The previous study on similar compounds conducted viability tests in normal fibroblasts, revealing selective cytotoxic properties in certain cancer cell lines [11].

Due to the significant antiproliferative activity observed for BA-TZ at 10 and 50 μM in melanoma cells, these concentrations were selected for DAPI staining in order to further investigate the compound’s more intimate cytotoxic mechanism. The effect and mechanism of TZ against melanoma cells have been scarcely investigated so far. Therefore, 10 and 50 μM of TZ were also tested in RPMI-7951 cells using the DAPI assay. Signs of apoptosis, such as nuclei condensation and shrinkage, were revealed for both compounds at 10 μM. The higher concentration, 50 μM, induced nuclear fragmentation, another sign of apoptosis. Apoptosis is the programmed cell death necessary for the natural removal of damaged cells, triggered by intracellular or cell surface signals. The process is clearly incriminated as a hallmark of cancer but also as responsible for tumor drug resistance [20]. Apoptosis was revealed as one of the several anticancer molecular mechanisms for betulinic acid [21] and other pentacyclic triterpenes [22] as well. Our research group previously synthesized C28-triazole derivatives of betulinic acid that were identified as apoptotic agents through the upregulation of the proapoptotic Bax gene and the downregulation of antiapoptotic Bcl-2 [23]. Other studies were performed as well but focused on the 1,2,3-triazole moiety [24] linked in various positions on BA’s triterpenic scaffold. The derivatives were found to increase ROS generation, activate caspases 3 and 9, increase Bax and Bad expression and decrease Bcl2 and Bcl-xl expression, thus revealing a mitochondria-dependent pathway apoptosis induction. The compound synthesized in the current study involves the 1,2,4-triazole moiety linked at C30 in the molecule of betulinic acid; similar compounds were obtained by substitution in the same position with 1,2,3-triazole by Sidova et al. [11] and Shi et al. [13]. The two studies did not investigate, however, the molecular mechanisms involved in the anticancer activity of the resulting derivatives. We may conclude that our results are in line with these previous studies but also reveal for the first time the apoptotic activity of C30 1,2,4-triazole derivatives of betulinic acid.

Mitochondria’s involvement in cancer development, growth and survival extends far beyond their energetic role; cancer cells are heavily influenced by oncoproteins/metabolites, ROS production, Ca^2+^ homeostasis, autophagy and apoptosis, all regulated by the mitochondria [25]. The Warburg effect is indeed an undisputable feature of numerous cancer cells and, until recently, the “injury to respiration” and consecutive glycolysis were widely accepted as cancer hallmarks. Recently emerged, the “metabolic plasticity” theory states that cancer cells can easily switch between OXPHOS and aerobic glycolysis, thus providing a mechanism that will lead to chemoresistance [26]. The dependance of cancer cells on OXPHOS has been described in solid tumors that have a poorly vascularized core that limits the glucose amount and oxygen availability, and, hence, it continues to use the mitochondrial electron transport system (ETS) for energy production (Le et al., 2014, Rumsey et al., 1990). Moreover, the last decade has brought much evidence about the involvement of mitochondrial metabolism in isolated cells that currently challenges the Warburg effect [27,28]. The dependence of Hodgkin-Reed-Sternberg (HRS) cells on mitochondrial OXPHOS has been demonstrated by Birkenmeier et al. [29]; the authors demonstrated that the HRS cells exhibited a pronounced dependency on OXPHOS energy conversion compared to normal germinal center (GC) B cells and showed that OXPHOS inhibitors could have a therapeutic utility in the treatment of classical Hodgkin lymphoma. Similar findings were reported both on mitochondria isolated from Diffuse Large B-Cell Lymphoma cell lines and on isolated cells, respectively [30]. Another study provided the first functional in vivo evidence that showed the upregulation of OXPHOS in epithelial tumor cells, reporting it as a common feature of human breast cancers [31]. High levels of OXPHOS were reported also in human breast carcinoma cell lines MDA-MB-468, MCF-7 and MDA-MB-231 cells, where the pharmacological inhibition of OXPHOS led to the decrease of cancer cell proliferation, migration capacity and invasiveness [32]. BRAF mutations are the most common alterations found in melanoma [33]. However, despite their proven efficacy, it was demonstrated that treatment of melanomas with BRAF inhibitors renders them dependent on ATP generation by the mitochondria and the association with inhibitors of oxidative phosphorylation may enhance the effect of BRAF inhibitors in melanoma patients [33]. On a similar note, and as supportive evidence, inhibition of mitochondrial oxidative phosphorylation by phenformin associated with BRAF inhibitor PLX4720 was able to induce tumor regression in a genetically engineered BRAFV600E/PTEN null-driven mouse model of melanoma and in nude mice bearing melanoma xenografts [34]. A switch to OXPHOS was reported as the main feature of platinum-resistant ovarian cancer stem cells [35]. In order to elucidate the mechanism of chemoresistance in triple-negative breast cancer, Lee et al. [36] reported that breast chemotherapy-resistant cancer stem cells rely on OXPHOS, and its activation is induced by increased MYC (a proto-oncogene that encodes a transcription factor) and MCL1 (an antiapoptotic Bcl-2 family protein) levels. A high OXPHOS status was also described in therapy-resistant acute myeloid leukemia cells, which rendered them vulnerable to OXPHOS inhibition strategies [37].

The extended SUIT protocol was applied to permeabilized normal HaCaT and malignant melanoma RPMI-7951 cells, following a 24 h treatment period with 10 μM BA-TZ. Mitochondrial respiratory rates measured at 37 °C revealed that BA-TZ at 10 μM can significantly inhibit the mitochondrial function of RPMI-7951 cells without affecting normal HaCaT cells (Figure 5 and Figure 6). In detail, the first mitochondrial rate decreased by BA-TZ was the routine respiration that depends only on the endogenous available ADP. The cell membrane was afterwards permeabilized using digitonine, for the purpose of evaluating the extended functional oxidative phosphorylation (OXPHOS) and to allow the passage of soluble molecules between the external media and cytosol. The subsequent basal, as well as LEAK, respiration were also significantly decreased as a result of BA-TZ treatment, thus suggesting that this compound can decrease the non-phosphorylating resting state of intrinsic uncoupled or dyscoupled respiration. BA-TZ at 10 μM can significantly inhibit the active respiration dependent both on complex I alone (OXPHOS_CI_) and on complex I + complex II (OXPHOS_CI+II_), respectively (Figure 5 and Figure 6). By titrating precise amounts of FCCP, a classical uncoupler of mitochondrial respiration, one can measure the maximal respiratory capacity of the electron transport system (dependent on both complex I and II) in the fully noncoupled state (ETS_CI+II_); subsequent inhibition of CI with rotenone allows the measuring of the ETS dependent solely on complex II. Upon evaluating the outer mitochondrial membrane integrity by Cyt c addition, BA-TZ revealed the ability to increase Cyt c, suggesting its ability to impair the outer mitochondrial membrane integrity of a malignant melanoma RPMI-7951 cell. This test, however, does not demonstrate that BA-TZ directly causes its permeabilization but rather advocates that BA-TZ indirectly, through an indirect mechanism of action, may lead to loss of mitochondrial outer membrane (mtOM) integrity. This effect is supported by BA and several of its 1,2,3-triazole derviatives’ capacity to increase Bax expression and decrease Bcl2 expression [12,38]. In turn, it is known that the antiapoptotic Bcl-2 acts as keeper of the mtOM integrity by suppressing the activity of proapoptotic Bax, which promotes the opening of the mitochondrial outer membrane permeabilization pore (MOMP), followed by apoptosis initiation [39]. Additionally, the observed decrease in State2_CI_ and State4_CI+CII_ (basal and LEAK respiration) indicates that BA-TZ produces both a decrease in the resting state oxygen consumption, when ATP synthase is unactive, and a decrease in proton transport across the inner mitochondrial membrane. According to previous research, this mechanism is the precise opposite of mitochondrial uncoupling, which is characterized by increased H^+^ transfer across the inner mitochondrial membrane [40]. While uncoupling has been shown to reduce ROS [41], these findings suggest that BA-TZ may also increase ROS production. These findings are similar to those reported by Coricovac et al. [42], where betulinic acid alone was able to inhibit mitochondrial function of A375 melanoma cells. More specifically, at the same concentration (10 μM), betulinic acid alone significantly decreased all respiratory rates: routine, basal, active, leak and maximal uncoupled respiration. A similar behavior on mitochondrial function was described on the same A375 melanoma cell line upon testing 1-hydroxybenzotriazole esters of betulinic, oleanolic and ursolic acid. All tested compounds were able to inhibit active respiration and decrease the maximal respiratory capacity and thus to induce mitochondrial dysfunction and trigger cell death [23]. As importantly pointed out by these authors, mitochondrial dysfunction and impairment of ATP production is a benefic effect in cancer cells, and all compounds that can induce such changes at the mitochondrial level and mitochondrial alterations can be considered promising therapeutic tools for cancer [23,42]. Furthermore, given the structural and targeted mitochondrial respiration similarities between BA-TZ and previously studied BA and BA triazole derivatives, we can conclude that, in addition to the above-mentioned action, our compound may induce apoptosis by disrupting the Bcl2/Bax ratio. This hypothesis, however, requires further investigation.

## 4. Materials and Methods

### 4.1. Reagents

All reagents used for chemical experiments, ethyl acetate, chloroform (CHCl_3_), tetracarbon chloride (CCl_4_), *N,N*-dimethylformamide (DMF), thiosemicarbazide, formic acid, potassium carbonate, sodium hydroxide, hydrochloric acid, betulinic acid (BA), *N*-bromo-succinimide (NBS) and 5-Fluorouracil (5-FU), were commercially purchased from Sigma Aldrich (Merck KGaA, Darmstadt, Germany) and were further employed without additional purification. For the in vitro experiments, the following reagents were utilized: dimethyl sulfoxide (DMSO-solvent), specific culture media: Dulbecco’s Modified Eagle’s Medium (DMEM) and Eagle’s Minimum Essential Medium (EMEM), cell culture supplements—fetal bovine serum (FBS) and penicillin/streptomycin (Pen/Strep); trypsin-EDTA solution, phosphate saline buffer (PBS), Trypan blue and 3-(4,5-dimethylthiazol-2-yl)-2,5-diphenyltetrazolium bromide (MTT) viability kit were acquired from Sigma Aldrich, Merck KGa Group (Darmstadt, Germany). All reagents used were analytically pure and appropriate for cell culture use.

### 4.2. Chemistry

#### 4.2.1. Instruments

The NMR spectra have been recorded on a Bruker NEO 400 MHz Spectrometer (Bruker, Billerica, MA, USA) recorded in DMSO-*d_6_* or CDCl_3_, at room temperature, using the standard parameter sets as provided by Bruker and were calibrated on the solvent residual peak (^1^H: 2.51 ppm for DMSO-*d_6_* or 7.26 ppm for CDCl_3_; ^13^C: 39.5 ppm for DMSO-*d_6_* or 77.0 for CDCl_3_). The peaks from the ^1^H NMR and ^13^C NMR spectra were assigned using information obtained from additional NMR experiments: ^13^C-DEPT135 and 2D NMR experiments (H,H-COSY; H,C-HSQC, H,C-HMBC). FTIR spectra were recorded using KBr pellets, on a Shimadzu IR Affinity-1S spectrophotometer in a 400–4000 cm^−1^ range and a 4 cm^−1^ resolution. Melting points were recorded using a Biobase melting point apparatus (Biobase Group, Shandong, China); thin-layer chromatography (TLC) was achieved using 60 F254 silica gel-coated plates (Merck KGaA, Darmstadt, Germany). LC/MS analysis was achieved using an Agilent 6120 Quadrupole LC/MS system (Santa Clara, CA, USA) equipped with a UV detector, ESI ionization source, and a Zorbax Rapid Resolution SB-C18 (1.8 μm; 50 mm × 2.1 mm) column. LC/MS spectra of all samples were carried out using methanolic solutions of the compounds tested. All samples were analyzed using an 85% methanol:15% ammonium formate 1 mM mixture as an isocratic mobile phase, at a flow rate of 0.4 mL/min, 25 ˚C, and l = 250 nm. Mass spectra were recorded in the negative ion mode using optimized ESI parameters.

#### 4.2.2. Synthesis Procedure for 1*H*-1,2,4-Triazole-3-thiol (TZ)

The protocol for obtaining 1,2,4-triazole-3-thiol (TZ) was implanted following previous reported methods [43] and required a 2-step reaction process. Firstly, 1-formyl-3-thiosemicarbazide was obtained by boiling 20 mL formic acid 90% (≈0.5 moles) with 9.1 g of thiosemicarbazide (0.1 moles) for 30 min, during which 1-formyl-3-thiosemicarbazide starts to crystalize. 30 mL of cold water was added, and the obtained emulsion was filtered and kept in an ice bath in order to let 1-formyl-3-thiosemicarbazide crystalize. The obtained crystals were vacuum-filtered, oven-dried, and used without further purification. For the next step, a 50 mL round-bottom flask equipped with a magnetic stirrer was loaded with 1.2 g NaOH (30 mmoles), 20 mL H_2_O and, after NaOH dissolution, 3.35 g 1-formyl-3-thiosemicarbazide (28.1 mmoles) and was refluxed for 1 h. After the reaction was completed, the reaction was cooled and the final product was precipitated with concentrated HCl and filtered.

*1H-1,2,4-triazole-3-thiol (TZ);* white powder, m.p. 221–222 °C, yield 68%; ^1^H NMR (400.13 MHz, DMSO-*d_6_*, δ, ppm): 13.51 (s, 1H, SH), 13.29 (s, 1H, NH), 8.26 (d, *J* = 1.1 Hz, 1H, H3); ^13^C NMR (100.6 MHz, DMSO-*d_6_*, δ, ppm): 165.5 (C5), 140.5 (C3); FTIR [KBr] (cm^−1^) relevant peaks: 3072 (N-H stretch); 2949, 2862 (C-H stretch); 2613 (S-H stretch); 1556 (-N=N stretch). LC-MS *R*t = 0.423 min, *m/z* = 100.0 [M-H^+^]^−^.

#### 4.2.3. Synthesis Procedure for 3β-*O*-Acetyl-30-bromobetulinic Acid

BA acetylation was done using a modified previously described method [44] and was achieved by reacting BA (1 Eq) with acetic anhydride (4 Eq) in pyridine, in the presence of DMAP (0.1 Eq), at room temperature for 12 h. The reaction mixture was diluted with water and extracted with CHCl_3_ 3 times. The organic phase was dried with anhydrous MgSO_4_, after which the solvent was removed using a rotary evaporator. The obtained 3β-*O*-Acetyl-BA was further used without additional purification. Next, 2.5 g of acetylated BA (≈5 mmoles) was dissolved in 50 mL CCl_4_, after which 1.78 g of freshly recrystallized NBS (10 mmoles) was added. The reaction was kept at room temperature for 48 h, after which the solution was filtered, the solvent was evaporated and the crude product was chromatographed over silica using a CHCl_3_: ethyl acetate mixture of 40:1 volume ratio. Obtained spectral data were in accordance with the literature [11].

3β-*O*-Acetyl-30-bromobetulinic acid; colourless crystalline powder, m.p. 284–290 °C, yield 60%; ^1^H NMR (400.13 MHz, CDCl_3_, δ, ppm): 11.07 (s, 1H, COOH), 5.15 (s, 1H, H29a), 5.05 (s, 1H, H29b), 4.46 (dd, *J* = 6.1 Hz, *J* = 10.0 Hz, 1H, H3), 4.02 (AB spin system, *J* = 10.5 Hz, 2H, H30), 3.03 (td, *J* = 4.4 Hz, *J* = 11.2 Hz, 1H, H19), 2.31 (d, *J* = 12.4 Hz, 1H, H21a), 2.22–2.15 (m, 2H, H1), 2.04 (s, 3H, H32), 1.97 (dd, *J* = 7.9 Hz, *J* = 12.4 Hz, 1H, H22a), 1.79–1.35 (m, H1a, H2a, H6, H7, H11a, H12, H13, H15a, H16, H18, H21b, H22b), 1.28–1.09 (m, H2b, H9, H11b, H15b), 0.98–0.92 (m, H1b, H23, H26, H27), 0.85–0.77 (m, H5, H24, H25). ^13^C NMR (100.6 MHz, CDCl_3_, δ, ppm): 181.9 (C28), 171.1 (C31), 151.2 (C20), 113.5 (C29), 80.9 (C3), 56.4 (C17), 55.4 (C5), 50.8 (C18), 50.4 (C9), 43.0 (C19), 42.4 (C14), 40.7 (C8), 38.4 (C1, C13), 37.8 (C4), 37.1 (C30), 36.8 (C10, C22), 34.3 (C7), 33.1 (C16), 32.0 (C21), 29.7 (C15), 27.9 (C23), 26.8 (C2), 23.7 (C12), 21.3 (C32), 21.0 (C11), 18.1 (C6), 16.5 (C24), 16.2 (C27), 16.1 (C25), 14.7 (C26). FTIR [KBr] (cm^−1^) relevant peaks: 2949, 2862 (C-H stretch); 1734, 1689 (ester and carboxyl C=O stretch); LC.MS *R*t = 4.23 min, *m/z* = 576 [M-H^+^]^−^.

#### 4.2.4. Synthesis Procedure for 3β-*O*-Acetyl-30-(1*H*-1,2,4-triazol-3-ylsulfanyl)-betulinic Acid

A round-bottom flask equipped with a magnetic stirrer was loaded with 5 mL DMF, 0.3 mmoles of dry K_2_CO_3_, 0.2 mmoles 1,2,4-triazole-3-thiol and, finally, 0.2 mmoles of 3β-*O*-Acetyl-30-bromobetulinic acid. The reaction was stirred at room temperature for 72 h. After completion, 50 mL of water was added, and the formed suspension was extracted with CHCl_3_ (4 ×1 5 mL). The organic phase was dried over anhydrous MgSO_4_, the solvent was evaporated and the crude product was chromatographed over silica using a CHCl_3_: ethyl acetate mixture of 1:1, volume ratio.

3β-*O*-Acetyl-30-(1*H*-1,2,4-triazole-3-ylsulfanyl)-betulinic acid (BA-TZ); white crystalline powder, m.p. 142–150 °C, yield 25%; ^1^H NMR (400.13 MHz, CDCl_3_, δ, ppm): 8.65 (s, 1H, H34), 7.55 (br s, 2H, NH, COOH), 5.07, (s, 1H, H29a), 4.97 (s, 1H, H29b), 4.45 (dd, *J* = 4.4 Hz, *J* = 9.8 Hz, 1H, H3), 3.91 (AB spin system, *J* = 13.6 Hz, 2H, H30), 3.01 (td, *J* = 4.4 Hz, *J* = 10.4 Hz, 1H, H19), 2.28 (d, *J* = 11.5 Hz, 1H, H21a), 2.15–2.10 (m, 2H, H1), 2.04 (s, 3H, H32), 1.95–1.90 (m, 1H, H22a), 1.72 (t, *J* = 11.2 Hz, 1H, H18), 1.61–1.16 (m, H1a, H2a, H6, H7, H11a, H12, H13, H15a, H16, H21b, H22b), 1.05–0.75 (m, H1b, H2b, H5, H9, H11b, H15b, H23, H24, H25, H26, H27). ^13^C NMR (100.6 MHz, CDCl_3_, δ, ppm): 180.7 (C28), 171.3 (C31), 155.8 (C33), 149.4 (C20), 146.2 (C34), 112.2 (C29), 81.1 (C3), 56.4 (C17), 55.4 (C5), 50.7 (C18), 50.3 (C9), 43.6 (C19), 42.4 (C14), 40.7 (C8), 39.3 (C30) 38.4 (C1, C13), 37.8 (C4), 37.0 (C10), 36.7 (C22), 34.3 (C7), 32.8 (C16), 32.0 (C21), 29.6 (C15), 27.9 (C23), 27.1 (C2), 23.7 (C12), 21.3 (C32), 21.0 (C11), 18.2 (C6), 16.5 (C24), 16.2 (C27), 16.0 (C25), 14.7 (C26). FTIR [KBr] (cm^−1^) relevant peaks:3132 (N-H stretch); 2945, 2872 (C-H stretch); 1710, 1641 (ester and carboxyl C=O stretch); LC-MS *R*t = 2.32 min, *m/z* = 596 [M-H^+^]^−^.

### 4.3. Cell Lines and Cell Culture Conditions

The cell lines selected for this study, HaCaT—immortalized human keratinocytes, were purchased from CLS Cell Lines Service GmbH (Eppelheim, Germany), and RPMI-7951—human malignant melanoma cells (ATCC^®^ HTB-66™) were procured from American Type Culture Collection (ATCC, Lomianki, Poland). DMEM, containing 10% FBS and 1% Pen/Strep mixture (10,000 IU/mL), was used to culture healthy skin cells. RPMI-7951 cells were grown in EMEM supplemented with 10% fetal bovine serum and 1% antibiotic mixture. The experiments were performed under standard conditions: incubation at 37 °C in 5% CO_2_ atmosphere.

### 4.4. Cellular Viability

The viability of the cells was evaluated using the MTT Blue assay. In brief, the cells were seeded in 96-well plates at a density of 1 × 10^4^ cells/well and stimulated with increasing concentrations (0.08–50 μM) of tested compound solubilized in DMSO (0.5%). After 24 h, the plates were incubated at 37 °C for 3 h with 10 µL/well of MTT reagent, followed by addition of 100 µL/well of solubilization buffer and stored at room temperature and protected from light for 30 min. The absorbance measurements were carried out at 570 nm using a xMark™ Microplate Spectrophotometer, Bio-Rad.

### 4.5. Mitochondrial Respiration Assessment

High-resolution respirometry studies (Oxygraph-2k Oroboros Instruments GmbH, Innsbruck, Austria) were employed in order to assess the mitochondrial respiratory function at 37 °C. For the measurement of respiratory rates, a modified substrate-uncoupler-inhibitor titration (SUIT) protocol was used, as previously described by Petruș et al. [45]. The cells used in this experiment were cultured in T25 culture flasks until reaching 80–85% confluence and treated with BA-TZ (10 μΜ) for 24 h. Afterwards, the cells were washed with PBS, trypsinized, counted and resuspended (1 × 10^6^/mL cells) in mitochondrial respiration medium (MIRO5: taurine 20 mM, EGTA 0.5 mM, MgCl_2_ 10 mM, K-lactobionate 60 mM, d-sucrose 110 mM, 3 mM KH_2_PO_4,_ HEPES 20 mM and BSA 1 g/L, pH 7.1). Initially, at the beginning of the respirometric protocol, the cells were suspended in MIRO5 and the oxygen flux was allowed to stabilize for 15 min. After stabilization, the first respiratory rate was recorded (routine respiration). The SUIT protocol, with the mitochondrial substrates/uncopulers/inhibitors used and the subsequent mitochondrial respiratory rates obtained, was as follows:

(i) The addition of digitonine (35 μg/L × 10^6^ cells, a mild detergent used to permeabilise the cellular membrane) together with CI substrates: glutamate (10 mM) and malate (5 mM) → measurement of basal respiration (State2_CI_);

(ii) ADP (5 mM) addition → measurement of active respiration dependent on CI (OXPHOS_CI_);

(iii) Addition of succinate (10 mM)—a CII substrate → measurement of OXPHOS_CI+CII_;

(iv) Addition of cytochrome c (10 μM)—evaluation of mitochondrial membrane integrity (Cyt c);

(v) Addition of oligomycin (1 μg/mL)—a complex V inhibitor → measurement of LEAK respiration dependent on CI and CII (State4_CI+II_);

(vi) Successive titrations with P-(trifluoromethoxy) phenylhydrazone carbonyl cyanide—FCCP (1 μM/step) → maximal respiratory capacity of the electron transport system (ETS_CI+II_) measurement;

(vii) Addition of rotenone (0.5 μM)—an inhibitor of CI→ measurement of the maximal respiratory capacity of the electron transport system that dependents solely on CI (ETS_CI_);

(viii) Addition of antimycin A (2.5 μM)—an inhibitor of CIII → measurement of residual oxygen consumption (ROX). All the obtained values were corrected after ROX.

### 4.6. Immunofluorescence Assay

The 4, 6′-Diamidino-2-Phenylindole (DAPI) staining was used in order to evaluate the nuclear morphology and to analyse the nuclear changes indicative of apoptosis (i.e., nuclear shrinkage/fragmentation). The RPMI-7951 cells (1 × 10^6^ cells/well), stimulated for 24 h at 37 °C with TZ and BA-TZ (10, 50 μΜ), were fixed for 30 min with 4% paraformaldehyde and afterwards permeabilized with 2% Triton X-100 in PBS for another 30 min. After 3 washes with cold PBS, the cells were blocked with 30% FCS in 0.01% Triton X for 1h at room temperature. Afterwards, following another washing step with cold PBS, the cells were stained with DAPI (300 nM) in a dark chamber. The counterstained nuclei were analysed using the Olympus IX73 inverted microscope at 40x magnification (Olympus, Tokyo, Japan) and the CellSens V1.15 software.

### 4.7. Statistical Analysis

All results were expressed as means ± standard deviation and were analyzed using GraphPad Prism software v.6 (San Diego, CA, USA). Two-way analysis of variance (ANOVA), followed by Bonferroni’s multiple comparisons post-test, was used to determine the statistical difference between the data sets obtained for the measurement of cellular viability. Values with *p* < 0.05 were considered to have a statistically significant difference (* *p* < 0.05, ** *p* < 0.005 and *** *p* < 0.0001). The mitochondrial respiratory rates were statistically analysed using one-way ANOVA, followed by Tukey’s multiple comparisons post-test. Values with *p* < 0.05 were considered to have a statistically significant difference (* *p* < 0.05, ** *p* < 0.01 and *** *p*< 0.001).

## 5. Conclusions

The current work reported the synthesis and antimelanoma induced cytotoxicity of a novel BA derivative, 3β-*O*-Acetyl-30-(1*H*-1,2,4-triazole-3-ylsulfanyl)-betulinic acid. While the antiproliferative biological assessment of this compound clearly showed that C30 modulation using 1,2,4-triazole is clearly beneficial for its antimelanoma potential, the synthetic pathway chosen for this study produced modest yields. Nevertheless, 1,2,4-triazole derivatives of BA represent a promising scaffold for the design of heterocyclic triterpenoid antimelanoma agents. Furthermore, by correlating previously reported data on C30 BA-1,2,3-triazole conjugates, future prospective developments involve the modulation of BA’s C30 position with various 5-phenyl substituted -1,2,4-triazoles and biological assessment of these compounds as well.

## Data Availability

Not applicable.

## Supplementary Materials

The following supporting information can be downloaded at: https://www.mdpi.com/article/10.3390/molecules27227807/s1. Figure S1: 1H NMR spectra of compound TZ; Figure S2: ^13^C NMR spectra of compound TZ; Figure S3: 1H NMR spectra of compound 3β-*O*-Acetyl-30-bromobetulinic acid; Figure S4: ^13^C NMR spectra of compound 3β-*O*-Acetyl-30-bromobetulinic acid; Figure S5: 1H NMR spectra of compound BA-TZ; Figure S6: ^13^C NMR spectra of compound BA-TZ; Figure S7: ^13^C-DEPT135 NMR spectra of compound BA-TZ; Figure S8: H,C-HMBC spectra of compound BA-TZ; Figure S9: H,C-HSQC spectra of compound BA-TZ; Figure S10: H,H-COSY spectra of compound BA-TZ; Figure S11: FTIR spectra of compound TZ; Figure S12: FTIR spectra of compound 3β-*O*-Acetyl-30-bromobetulinic acid; Figure S13: FTIR spectra of compound BA-TZ.

## Sample Availability

Samples of the compounds are not available from the authors.
