# Supplementary material for: The C30-Modulation of Betulinic Acid Using 1,2,4-Triazole: A Promising Strategy for Increasing Its Antimelanoma Cytotoxic Potential
Source: Molecules. 2022 Nov 12;27(22):7807. doi: 10.3390/molecules27227807 (PMC9697306; doi:10.3390/molecules27227807)
Supplement: Supplementary file 1 [file molecules-27-07807-s001.zip › molecules-2012201-supplementary.pdf]

SUPPLEMENTARY MATERIAL

The C30-Modulation of Betulinic Acid Using 1,2,4-Triazole: A Promising Strategy for Increasing Its Antimelanoma Cytotoxic Potential

Gabriela Nistor [1,2], Marius Mioc [1,2,*], Alexandra Mioc [2,3], Mihaela Balan-Porcarasu [4], Roxana Racoviceanu [1,2], Alexandra Prodea [1,2], Andreea Milan [1,2], Roxana Ghiulai [2,5], Alexandra Semenescu [2,6], Cristina Dehelean [2,6] and Codruța Șoica [2,5]

1 Department of Pharmaceutical Chemistry, Faculty of Pharmacy, "Victor Babes" University of Medicine and Pharmacy Timisoara, Eftimie Murgu Square No. 2, 300041 Timişoara, Romania
2 Research Centre for Pharmaco-Toxicological Evaluation, "Victor Babes" University of Medicine and Pharmacy, Eftimie Murgu Sq., No. 2, 300041 Timisoara, Romania
3 Department of Anatomy, Physiology, Pathophysiology, Faculty of Pharmacy, Victor Babes University of Medicine and Pharmacy, 2nd Eftimie Murgu Sq., 300041 Timisoara, Romania
4 Institute of Macromolecular Chemistry 'Petru Poni', 700487 Iasi, Romania
5 Department of Pharmacology-Pharmacotherapy, Victor Babes University of Medicine and Pharmacy, 2nd Eftimie Murgu Sq., 300041 Timisoara, Romania
6 Department of Toxicology, Faculty of Pharmacy, "Victor Babes" University of Medicine and Pharmacy, Eftimie Murgu Sq., No. 2, 300041 Timisoara, Romania
* Correspondence: marius.mioc@umft.ro; Tel.: +40-256-494-604

Abstract: Cancer, in all its types and manifestations, remains one of the most frequent causes of death worldwide; an important number of anticancer drugs have been developed from plants, fungi and animals, starting with natural compounds that were later derivatized in order to achieve an optimized pharmacokinetic/pharmacological profile. Betulinic acid is a pentacyclic triterpenic compound that was identified as an anticancer agent whose main advantage consists in its selective activity, which ensures the almost total lack of cytotoxic side effects. Conjugates of betulinic acid with substituted triazoles, scaffolds with significant pharmacological properties, were synthesized and tested as anticancer agents in order to achieve new therapeutic alternatives. The current paper aims to obtain a C30-1,2,4-triazole derivative of betulinic acid simultaneously acetylated at C3 whose biological activity was tested against RPMI melanoma cells. The compound revealed significant cytotoxic effects at the tested concentrations (2, 10 and 50 μM) by significantly decreasing the cell viability to 88.3%, 54.7% and 24.5%, respectively, as compared to the control. The compound's testing in normal HaCaT cells showed a lack of toxicity, which indicates its selective dose-dependent anticancer activity. The investigation of its underlying molecular mechanism revealed an apoptotic effect induced at the mitochondrial level, which was validated through high-resolution respirometry studies.

__Contents__

Figure S1. 1H NMR spectra of compound **TZ**
Figure S2. 13C NMR spectra of compound **TZ**
Figure S3. 1H NMR spectra of compound 3β-*O*-Acetyl-30-bromobetulinic acid
Figure S4. 13C NMR spectra of compound 3β-*O*-Acetyl-30-bromobetulinic acid
Figure S5. 1H NMR spectra of compound **BA-TZ**
Figure S6. 13C NMR spectra of compound **BA-TZ**
Figure S7. 13C-DEPT135 NMR spectra of compound **BA-TZ**
Figure S8. H,C-HMBC spectra of compound **BA-TZ**
Figure S9. H,C-HSQC spectra of compound **BA-TZ**
Figure S10. H,H-COSY spectra of compound **BA-TZ**
Figure S11. FTIR spectra of compound TZ
Figure S12. FTIR spectra of compound 3β-*O*-Acetyl-30-bromobetulinic acid

Figure S13. FTIR spectra of compound **BA-TZ**

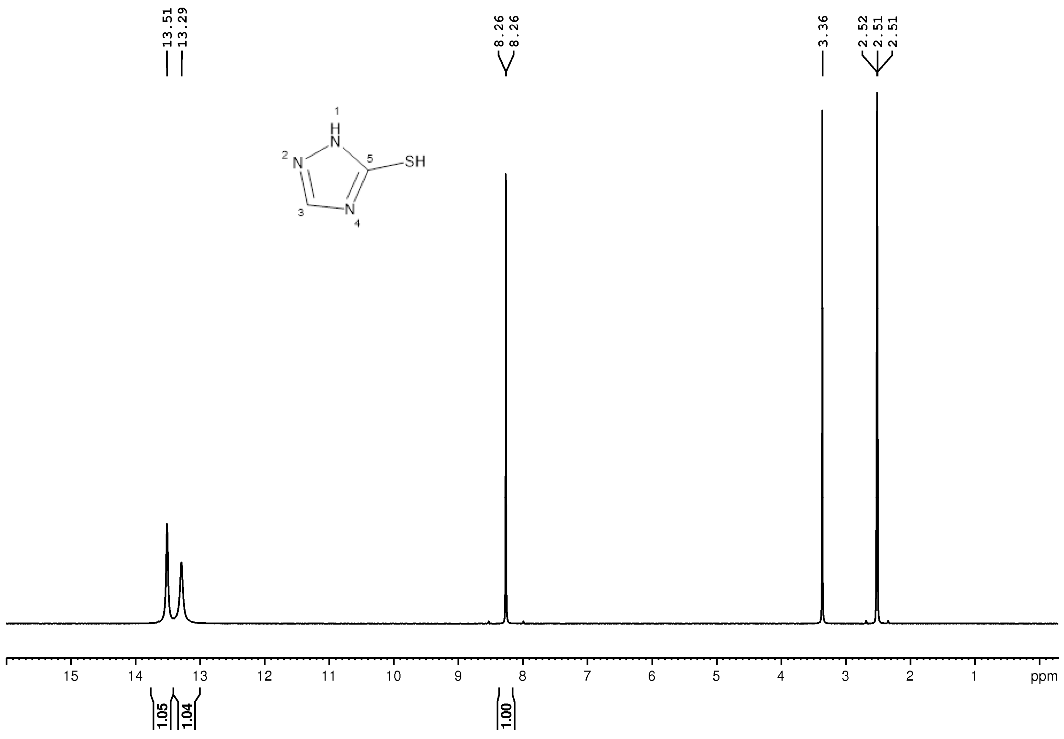

Figure S1. 1H NMR spectra of compound **TZ**

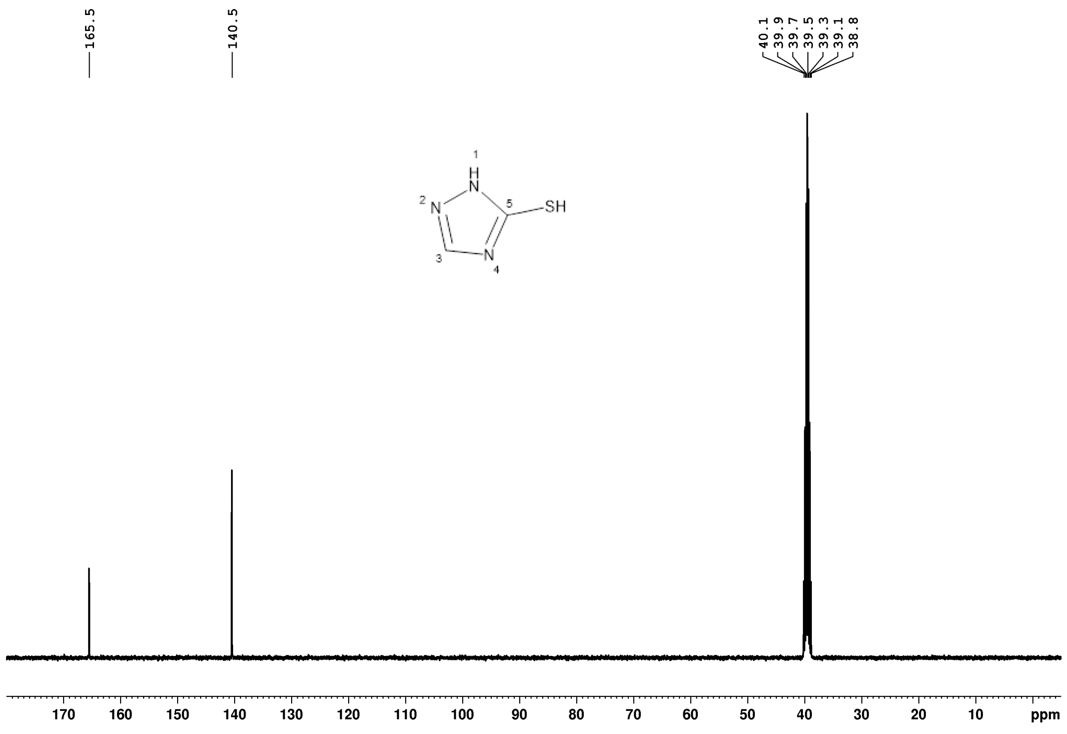

Figure S2. 13C NMR spectra of compound **TZ**

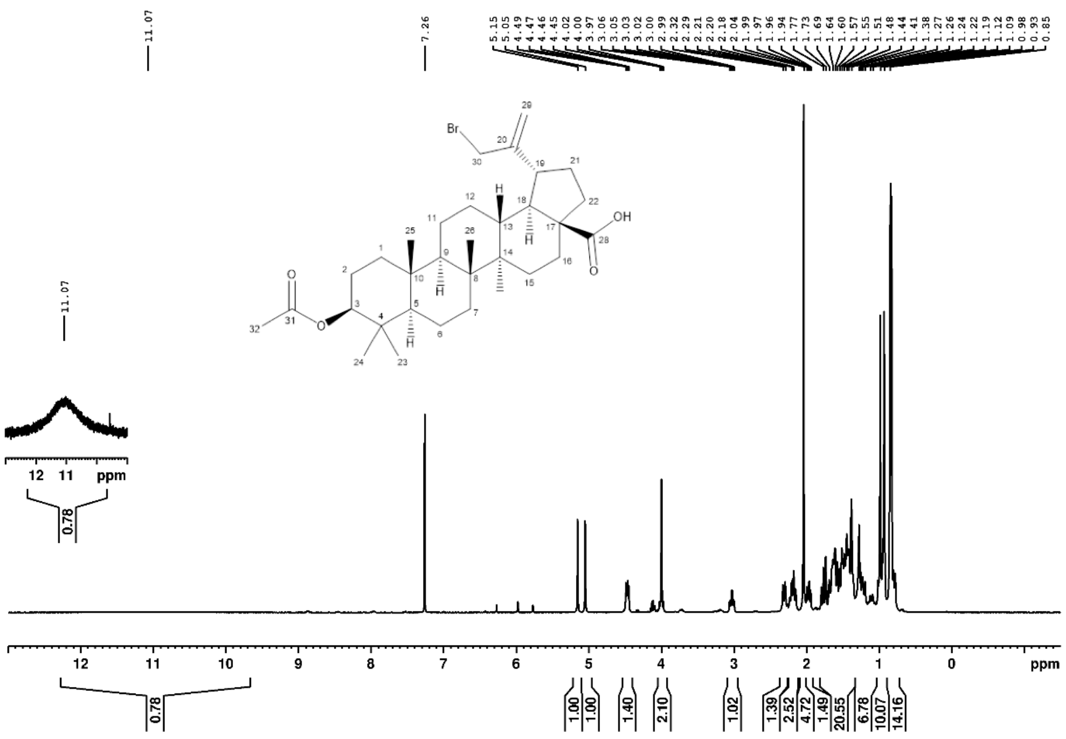

Figure S3. 1H NMR spectra of compound 3β-O-Acetyl-30-bromobetulinic acid

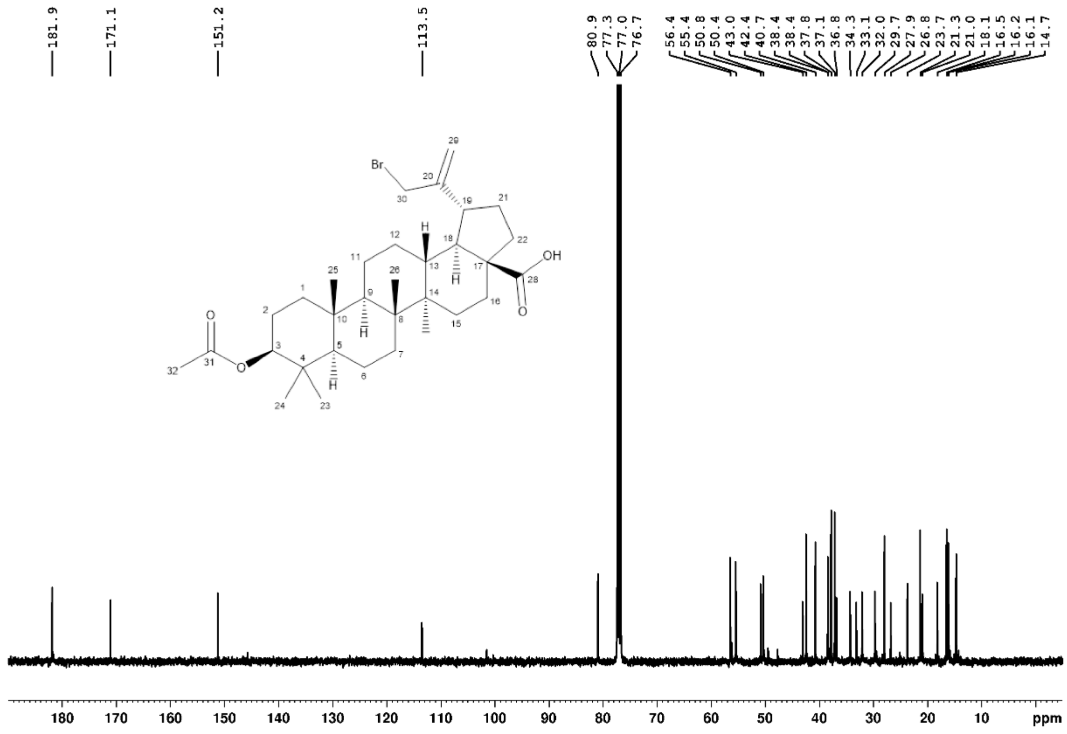

Figure S4. 13C NMR spectra of compound 3β-O-Acetyl-30-bromobetulinic acid

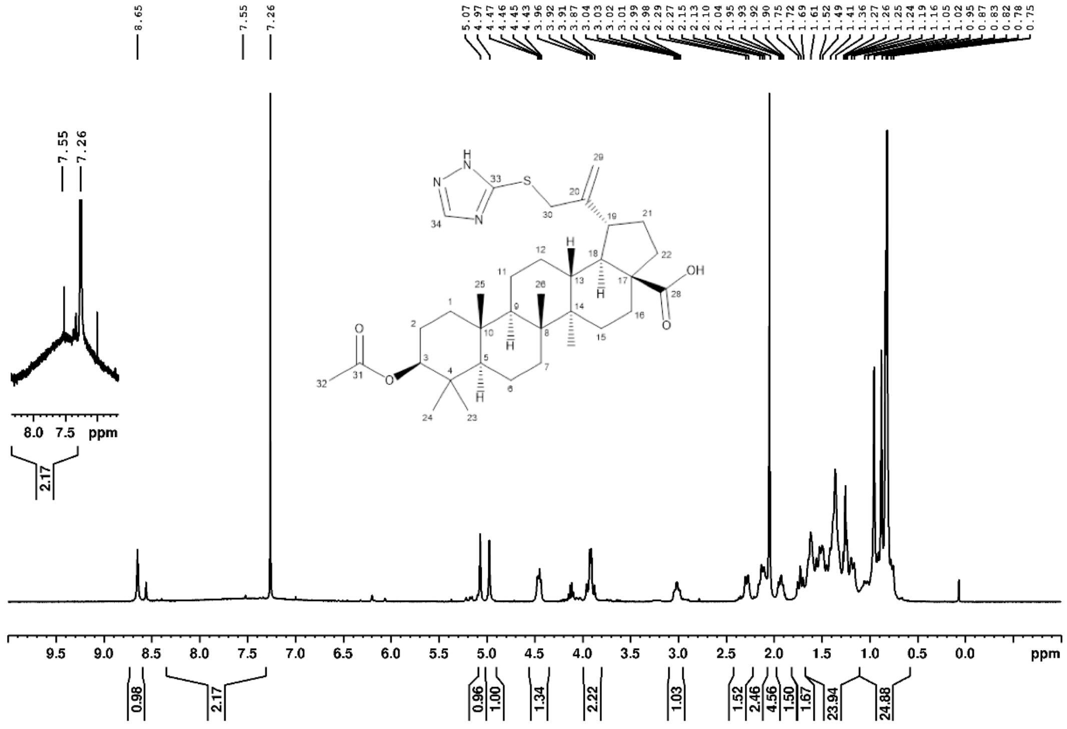

Figure S5. 1H NMR spectra of compound **BA-TZ**

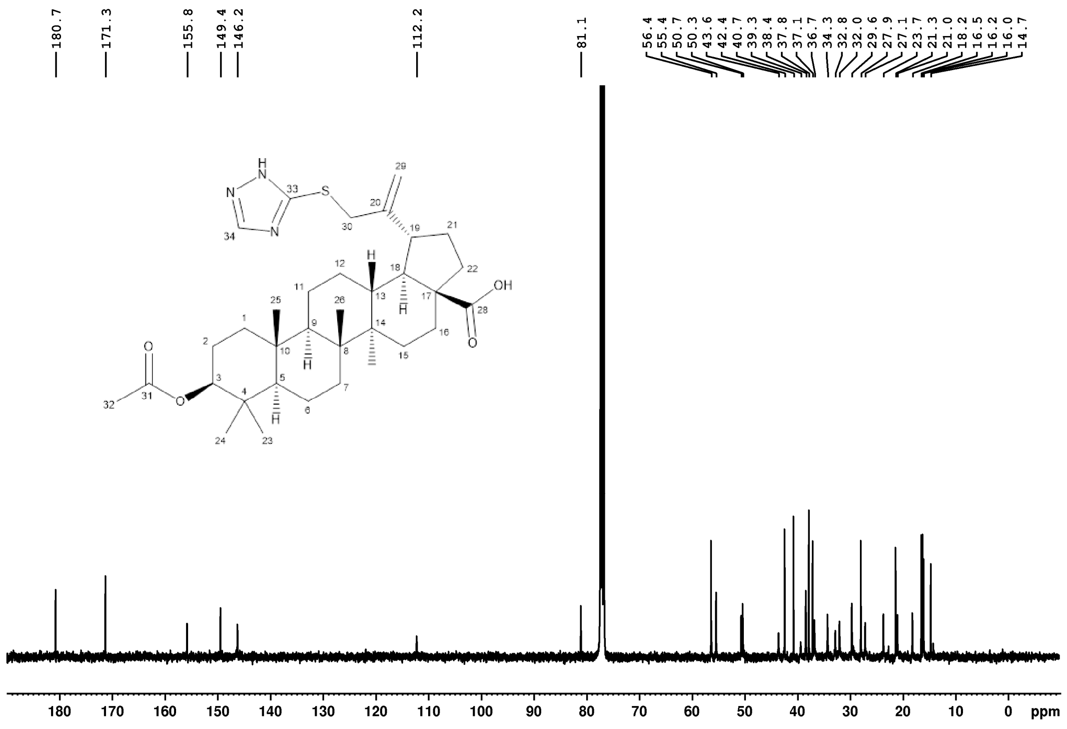

Figure S6. 13C NMR spectra of compound **BA-TZ**

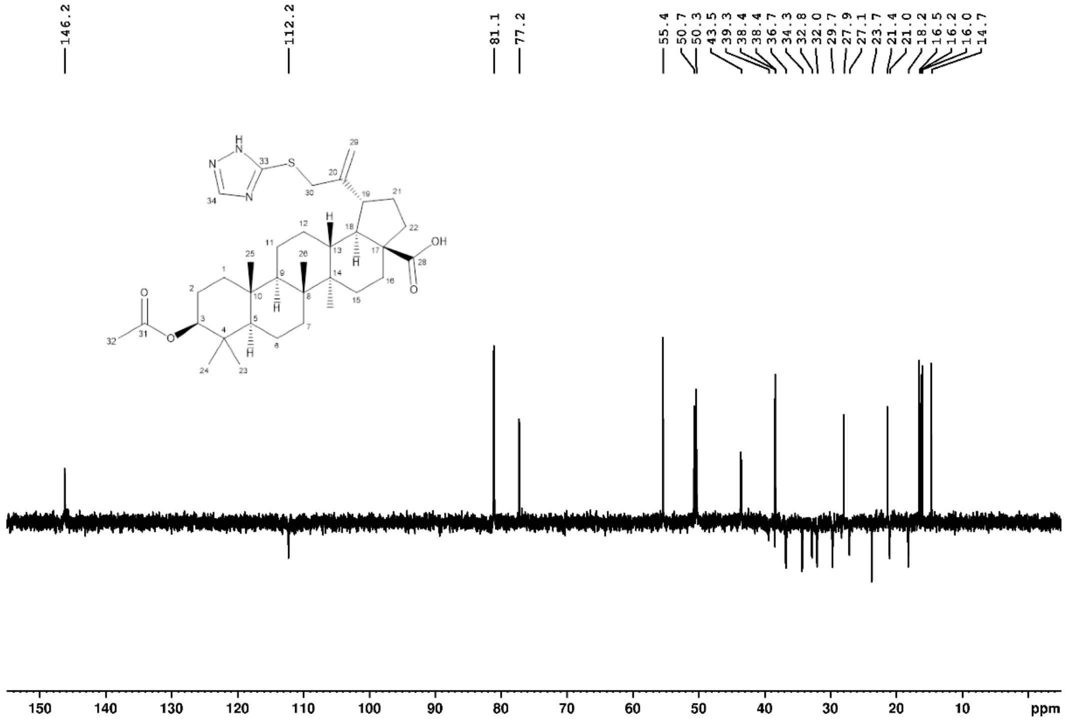

Figure S7. 13C-DEPT135 NMR spectra of compound **BA-TZ**

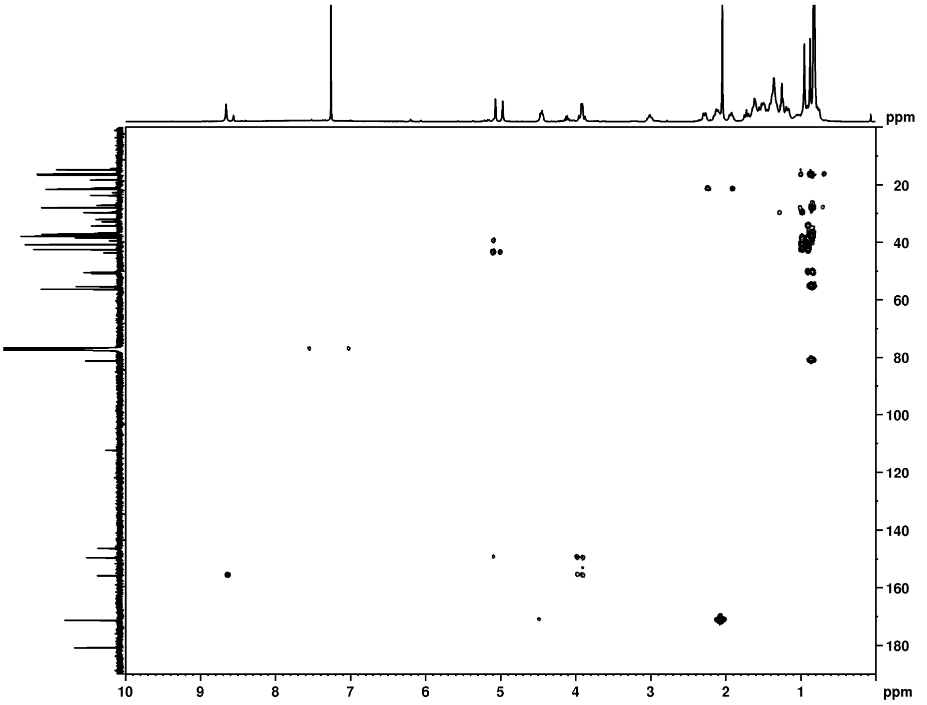

Figure S8. H,C-HMBC spectra of compound **BA-TZ**

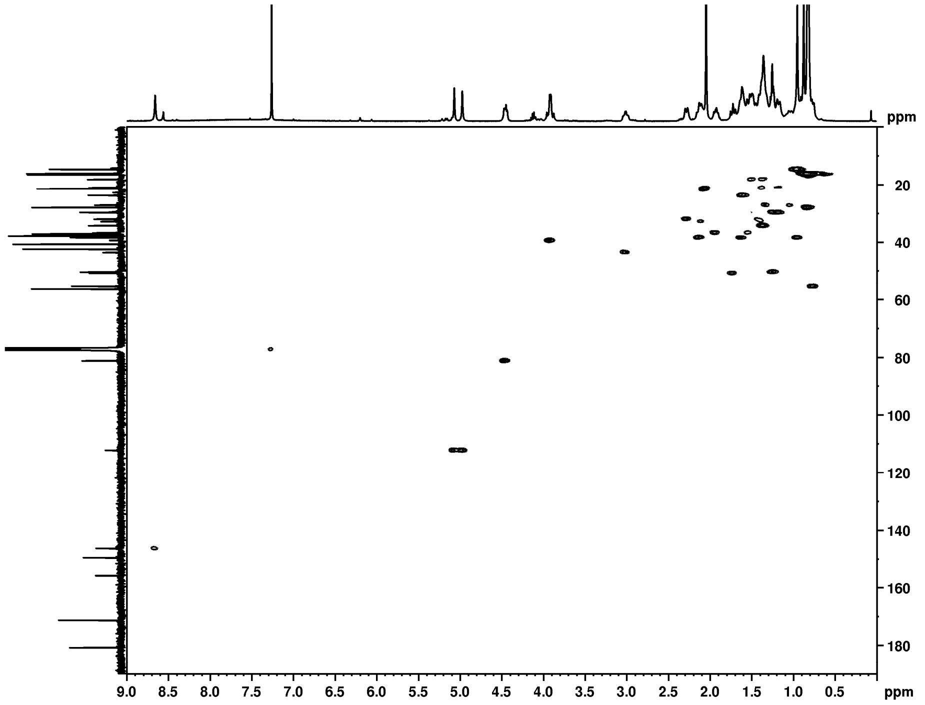

Figure S9. H,C-HSQC spectra of compound **BA-TZ**

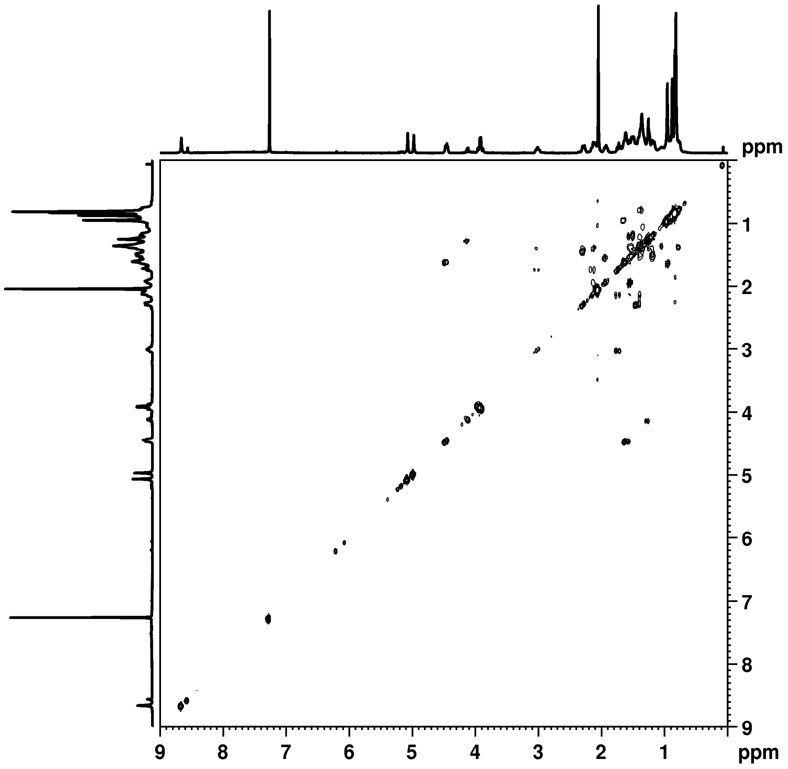

Figure S10. H,H-COSY spectra of compound **BA-TZ**

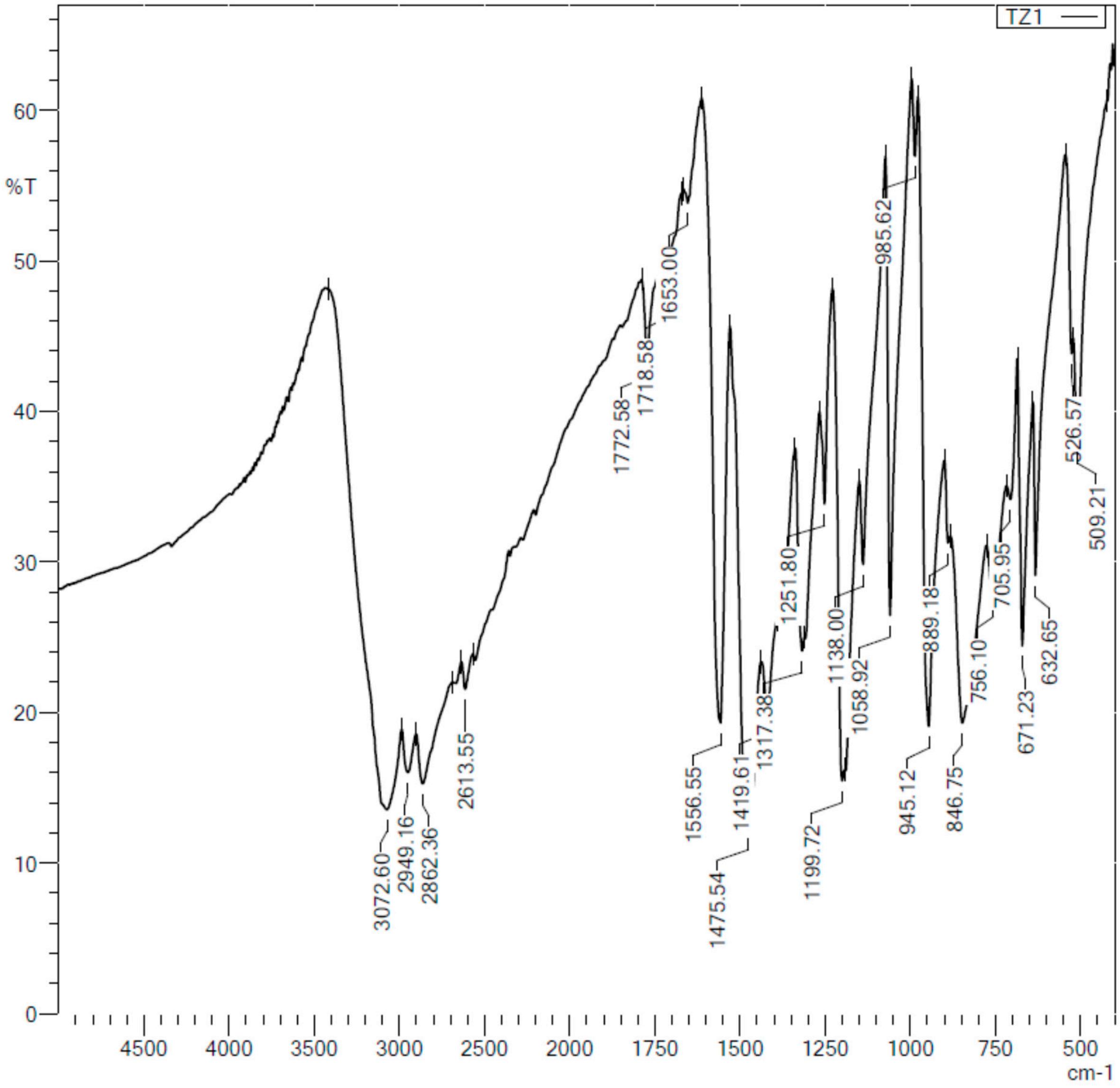

Figure S11. FTIR spectra of compound TZ

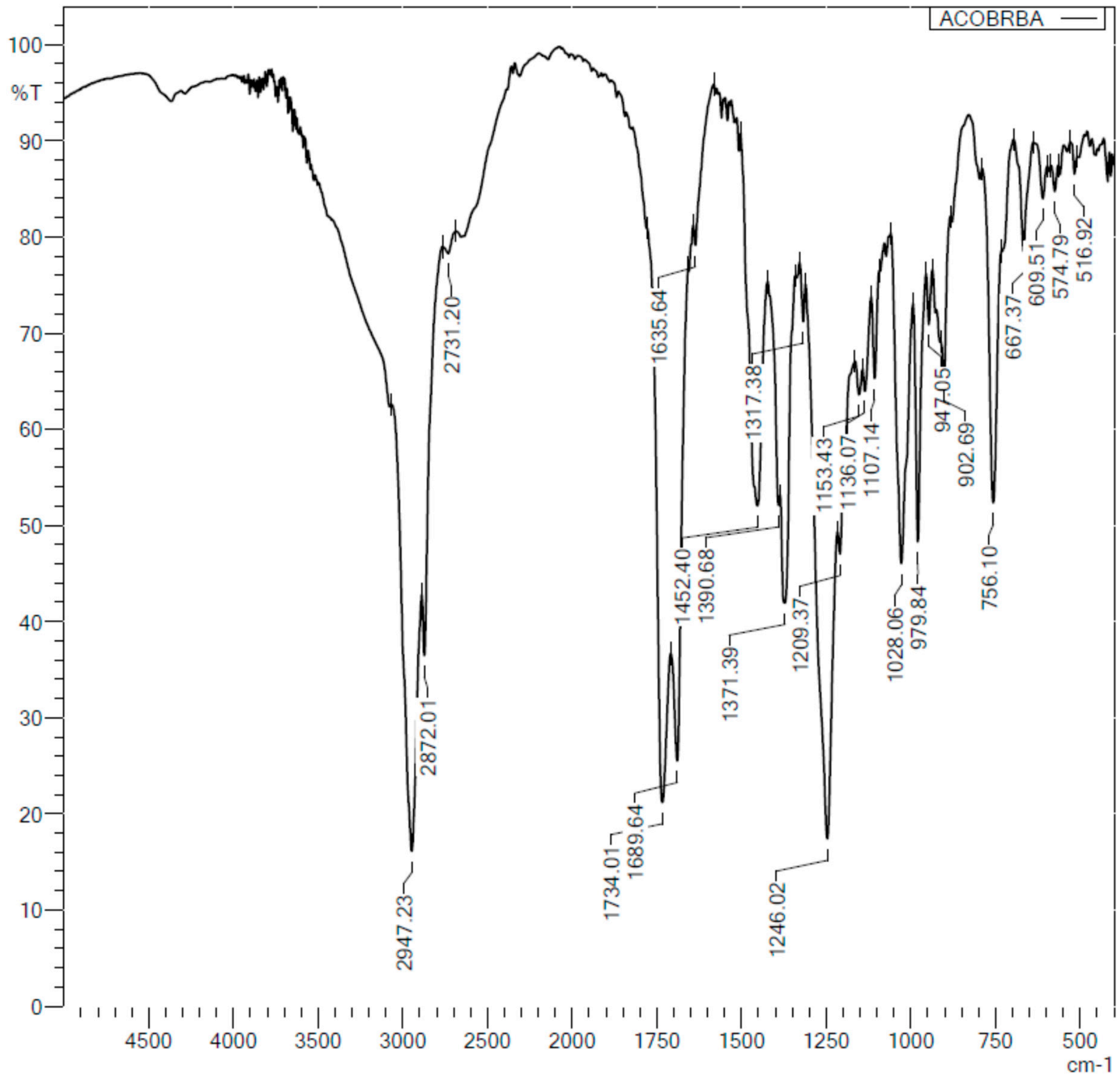

Figure S12. FTIR spectra of compound 3β-O-Acetyl-30-bromobetulinic acid

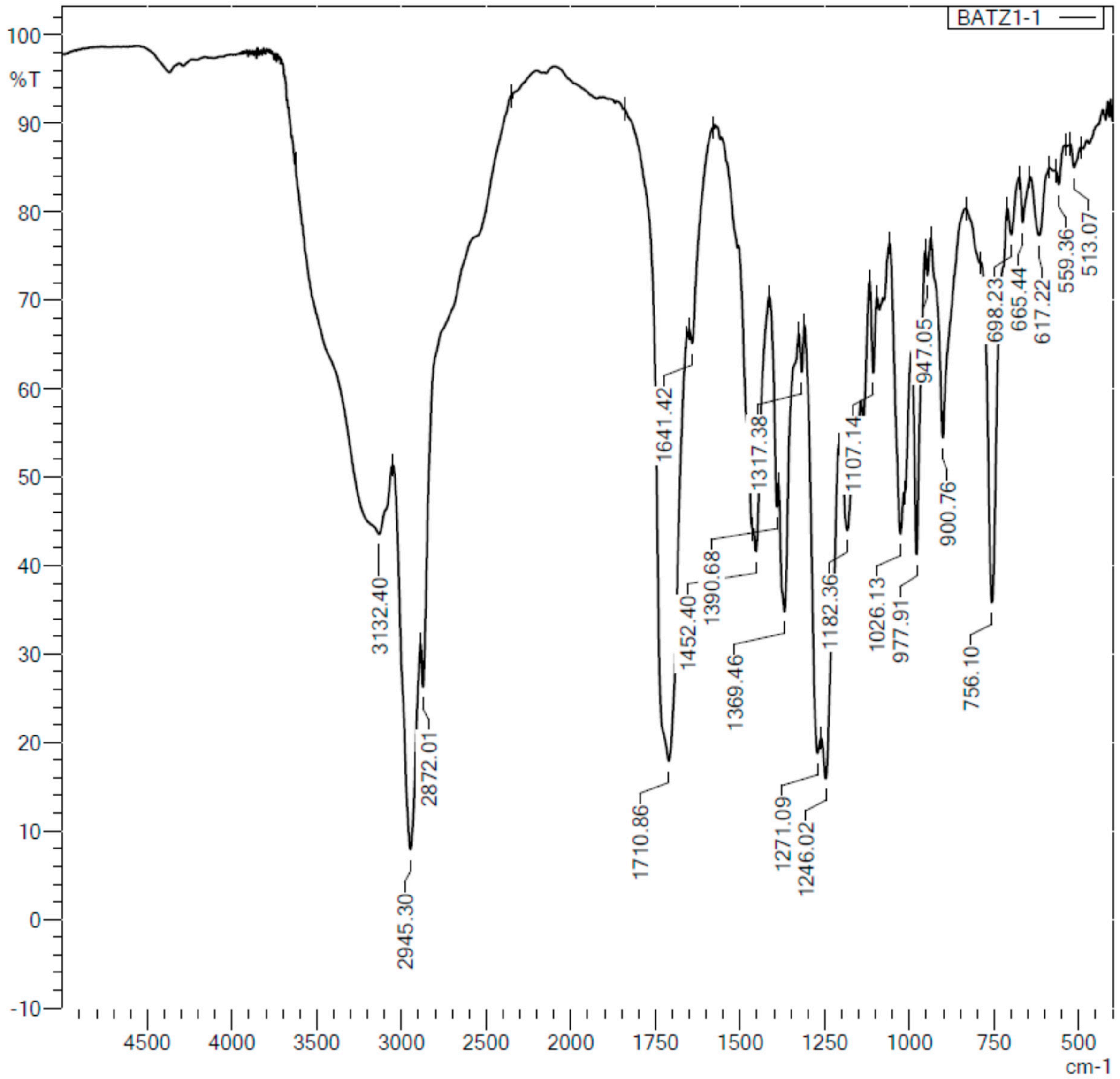

Figure S13. FTIR spectra of compound **BA-TZ**
